# The Prevalence and Causes of Visual Impairment in Type 2 Diabetes Mellitus in Northeast China

**DOI:** 10.1155/2020/5969816

**Published:** 2020-11-29

**Authors:** Liang Wen, Yu Wang, Zhong Lin, Feng Hua Wang, Xiao Xia Ding, Dong Li, Kemi Feng, Yuan Bo Liang, Dong Xiao Zhang, Yu Dou, Gang Zhai

**Affiliations:** ^1^Fushun Eye Hospital, Fushun, Liaoning, China; ^2^The Eye Hospital, School of Ophthalmology and Optometry, Wenzhou Medical University, Wenzhou, Zhejiang, China; ^3^Beijing Tongren Eye Center, Capital Medical University, Beijing Ophthalmology & Visual Science Key Lab, Beijing, China

## Abstract

**Purpose:**

To evaluate the prevalence and causes of visual impairment in a group of community people with type 2 diabetes mellitus (T2DM) in Northeast China.

**Methods:**

Population-based cross-sectional survey. Patients diagnosed with T2DM residing in 15 communities in Fushun, Northeast China, were enrolled between July 2012 and May 2013. All participants underwent an extensive and standardized eye examination (visual acuity testing, slit-lamp, and fundus examination). Low vision was defined as presenting VA of better-seeing eye <20/60 and ≥20/400, and blindness was defined as VA <20/400, according to the World Health Organization (WHO) definitions. The primary causes of blindness and low vision were assessed by senior ophthalmologists.

**Results:**

Visual acuity measurements were available for 1998 (89.8%) of 2224 subjects in the study. The prevalence of bilateral blindness and low vision defined was 0.90% and 10.81%. Uncorrected refractive error was the first leading cause of low vision (75.0%) and blindness (38.9%). After correcting the refractive error, the first leading cause of low vision was cataract (44.4%), followed by diabetic retinopathy (29.6%) and myopic maculopathy (18.5%), while the first leading cause of blindness was proliferative DR (45.4%), followed by cataract (36.4%) and myopic maculopathy (18.2%).

**Conclusions:**

This study suggested a high prevalence of low vision and blindness in this study cohort. Uncorrected refractive error and cataract remain the leading cause of visual impairment, but the major challenge is the early diagnosis and intervention of diabetic retinopathy to reduce diabetes-related blindness.

## 1. Introduction

The number of people living with diabetes mellitus (DM) is almost 4 times to 422 million adults since 1980, with most of them living in the developing countries. China has the largest number of people in the world with DM, approximately more than 109 million in 2015 and an estimation of 143 million by 2035 (http://www.idf.org/membership/wp/china). The National Health and Nutrition Survey conducted in the USA reported that visual impairment was more common in diabetics than in nondiabetics people [1]. Population-based studies have showed that persons with DM are prone to develop multiple ophthalmic conditions, including diabetic retinopathy (DR), macular edema (ME) [2, 3], cataract [4, 5], diabetic papillopathy [6], glaucoma [7], central retinal vein occlusion, dry eye syndrome, and infectious diseases [8]. These diseases are often associated with progressive visual loss. It was reported in the Los Angeles Latino Eye Study that the rate of visual impairment in persons with DM was 3 times higher (6%) than in those without (2%) [9]. In China, about 10 percent of the adults have DM, and the prevalence is even higher (greater than 20 percent) in the elderly population [10]. However, there is a paucity of population-based data on diabetic patients with visual impairment. The Fushun Diabetic Retinopathy Cohort Study (FS-DIRECT) is a community-based survey to determine the natural history of DR (incidence, progression, regression, etc.) in type 2 DM (T2DM). The purpose of this article was to report the prevalence and causes of visual impairment among this group of diabetic patients.

## 2. Materials and Methods

### 2.1. Study Design and Procedure

The FS-DIRECT is an ongoing community-based cohort study which completed the baseline recruitment between July 2012 and May 2013. Ethics committee approval was obtained from Fushun Eye Hospital. Written informed consent was obtained from all subjects. The details of the rationale, study design, and methodology of FS-DIRECT have been previously described [11]. Briefly, residents aged 30 years and above in Jiangjun Street, Fushun City, with T2DM were introduced to complete an extensive and standardized eye examination, a detailed questionnaire, and blood sample collection.

### 2.2. Visual Acuity Testing

The participants presenting VA was measured with whatever forms of current correction (e.g., glasses or contact lenses) that the participant might have worn at the time of the examination following the protocol of the Early Treatment Diabetic Retinopathy Study using the logMAR visual acuity chart (Precision Vision, USA) at 4 m. All the participants underwent automated refraction using the Automatic Refractor (NIDEK AR-610/630A, Japan). If the participant read fewer than 50 letters (Snellen equivalent of 20/25) at 4 m in either eye, the subjective refraction would be performed by a trained optometrist, and which was defined as the best-corrected visual acuity (BCVA). If the participant was unable to read 20 letters at 4 m (Snellen equivalent of 20/200), VA measurement was done at 1 m. If none of the letter could be recognized at a 1 m distance, VA was recorded as counting fingers [CF], hand movements [HM], light perception [LP], or no LP correspondingly. VA was scored as the total number of letters read correctly at 4 m or 1 m, and a logMAR score of 1.7 was designated to eyes with a VA of CF, HM, LP, or no LP [12].

### 2.3. Definition of Visual Impairment

Blindness and visual impairment were defined based on the World Health Organization criteria. Low vision was defined as presenting VA of the better-seeing eye <20/60 and ≥20/400, and blindness was defined as presenting VA of the better-seeing eye <20/400.

In order to compare our data with other studies, data by the definition of the United States (US) were also presented. Visual impairment was categorized as mild (20/40 ≤ BCVA ≤ 20/63), moderate (20/80 ≤ BCVA ≤ 20/160), and severe (BCVA ≤ 20/200). The severe visual impairment definition was also the blindness definition [12, 13].

In addition to reporting visual impairment in terms of the better-seeing eye, data on the worse-seeing eye based on the presenting and best-corrected VA were also presented.

### 2.4. Primary Causes of Visual Impairment

The primary causes of low vision and blindness were assessed according to the clinical history and ophthalmologic examination by two senior ophthalmologists. The main pathology for each person of the better eye was assigned as the cause of visual loss. If two or more diseases were present, the disease with the most significant and irreversible influence was assigned as the principal cause. Diabetic retinopathy: grading protocols for DR were modifications of the Early Treatment Diabetic Retinopathy Study (ETDRS) and adaptation of the modified Airlie House classification of DR [14]. Eyes were graded according to the following criteria: no DR (levels 10–20); nonproliferative DR (levels 31–53) (including mild nonproliferative [levels 31–37], moderate nonproliferative [levels 43–47], severe nonproliferative [levels 53]); and proliferative DR (levels 60–85). Diabetic macular edema (DME) was considered present if any area of the retina within 1 disc diameter from the center of the macula was thickened. Clinically significant macular edema (CSME) was diagnosed according to the ETDRS criteria or if there was a history of macular edema with evidence of photocoagulation treatment consistent with it. Cataract was regarded as the main cause of visual impairment based on the Lens opacities classification system III or the fundus was obscured by lens opacity. Uncorrected refractive error was defined as no visual impairment (improved to better than 20/60) after refraction and had visual impairment of 20/60 or worse before refraction. Myopic maculopathy was defined by a spherical equivalent of at least −6.0 diopters and the typical myopic maculopathy with stretching of the macula. Age-related macular degeneration (AMD) was characterized by hard distinct or soft distinct drusens with retinal pigment epithelial depigmentation or increased retinal pigment in the macular area with exudative AMD or geographic atrophy.

### 2.5. Statistical Analyses

Statistical analysis was performed using a commercially available statistical software package SAS 9.4 (SAS Institute Inc. Cary, NC, USA). The data were analyzed to determine the overall prevalence (95% confidential interval, CI) of blindness and visual impairment in the population of people with diabetes. The causes of visual impairment of main age groups were also ascertained.

## 3. Results

Two thousand and thirty three (91.4%) of 2224 subjects in this study and 1998 (89.8%) had visual acuity data. The majority of the subjects were female (59.2%). The mean age was 62.0 ± 9.0 years (range 33–91 years). The mean duration of DM was 7.7 ± 6.0 years (range 1 month to 40 years). Using the WHO standard definition, on presenting VA, the prevalence of bilateral blindness was 0.90% (95% CI, 0.53–1.42%) and bilateral low vision was 10.81% (95% CI, 9.48–12.26%). Using the US standard definition, it was 4.80% (95% CI, 3.91–5.84%) and 20.07% (95% CI, 18.33–21.89%), respectively. The corresponding prevalence of BCVA-defined blindness and low vision was 0.56% (95% CI, 0.28–1.00% WHO) and 2.76% (95% CI, 2.08–3.58% WHO); 1.22% (95% CI, 0.79–1.82% US) and 5.97% (95% CI, 4.96–7.11% US), respectively. Tables 1 and 2 also present the prevalence of visual impairment for unilateral eyes using the definition.

Based on presenting VA, the uncorrected refractive error was the first leading cause of low vision (75.0%) (Table 3). After refractive correction, cataract was the major cause of low vision (44.4%), followed by diabetic retinopathy (29.6%) and myopic maculopathy (18.5%). However, the first leading cause of blindness was proliferative DR (45.4%), followed by cataract (36.4%) and myopic maculopathy (18.2%) (Table 4). Records of both BCVA and DR grading were found for 3654 eyes of the 1998 patients. Among these eyes, 1382 (37.7%) eyes had DR.  Table 5 shows the frequency of visual impairment in each individual eye according to BCVA (WHO definition) in these 3654 eyes. In no DR, mild-to-moderate NPDR, severe NPDR, and PDR eyes, the prevalence of low vision was 3.65%, 5.5%, 7.0%, and 34.3% and the prevalence of blindness was 0.53%, 0.2%, 1.6%, and 15.3%. Among the eyes with photocoagulation or vitrectomy for PDR, 35.6% and 8.2% of the eyes were low vision and blindness, respectively.

## 4. Discussion

In this study, we provided essential baseline data on the prevalence of blindness and low vision in a defined population with type 2 diabetes. Our study showed a prevalence of low visual (2.76% WHO) and blindness (0.56% WHO) of BCVA defined in the population aged 30 years and above. In our region, the prevalence of low visual was similar to that of the Beijing Desheng community (2.5% WHO) which was also based on diabetic participants [15] and lower than that of the Handan Eye Study (4.2% WHO) with a small number of DM participants (387, 6.9%) [16] and another investigation on patients with diabetes mellitus from Tangshan, China (4.35% WHO) [17]. The prevalence of blindness was higher than that of the Beijing Desheng community (0.2%) and the Handan Eye Study (0.3%). Using the US definition (presenting VA), the prevalence of visual impairment in our study was 24.9% (20.1% mild and 4.8% severe), which was much higher compared with other studies from diabetic retinopathy screening programs [18–21]. In the Kwai Tsing district of Hong Kong, the prevalence of visual impairment was 11.3%, with 10.6% mild and 0.7% severe in T2DM [18]. In a cross-sectional analysis of the diabetic retinopathy in various ethnic groups in the UK (DRIVE-UK) study, with a total of 57,144 people diagnosed with diabetes, only 3.4% of people were visually impaired and 0.39% were severely visually impaired [19]. The UK has one of the most advantaged and quality assured DR screening programs in the world with a population coverage ranging from 80 to 95% (http://www.retinalscreening.nhs.uk). The Icelanders are also well screened for diabetes mellitus and have been offered with regular screening for DR since 1983. Therefore, loss of vision is uncommon in studies on DR from Iceland [20, 21]. It is well known that the incidence and prevalence of visual impairment is lower in populations with well-established screening for diabetic ocular disease than populations without screening.

The leading cause (75.0%) of bilateral presenting VA-defined low vision was uncorrected refractive error. This result was supported by studies performed in participants with or without diabetes in China. In the Beijing Desheng community, the major cause of low vision was also uncorrected refractive error (58.8%) based on diabetic participants [15]. In the Handan Eye study, 71.4% of the participants had no visual impairment (improved to better than 20/60) after refraction [22].

After refractive correction, cataract was the first leading cause of low vision (44.4%) and the second cause of blindness (36.4%). In the Beijing eye study, cataract was the major factor to the exponential increase for the prevalence of blindness and visual impairment in subjects aged 40 and above [23]. This was understandable due to the limited resource of refractive services and the lower cataract surgery rate (the CSR of year 2005, 2010, and 2012 is 440, 915, and 1072 in every million people per year separately in China compared to over than 9,000 in developed Western countries[http://www.moheyes.com/. The Cataract surgical rate should be improved first]). In our study sample of 2033 participants, 213 participants had cataract, but only 6 of them had undergone cataract surgery.

Diabetic retinopathy was the most common cause of blindness in this study due to vitreous hemorrhage or tractional retinal detachment and diabetic maculopathy. Similar results were reported in the Desheng community study and the Beijing study [15, 24]. But, in the surveys on regions with well-established DR screening, such as the UK and Sweden, the major cause of visual loss in a population with diabetes was not due to diabetic retinopathy [20, 25, 26]. In our study, there was no systematic screening service, and as a result 82.2% (175/213) of the diabetic patients with vision-threatening DR had not been treated. In our study, 73 eyes had undergone laser photocoagulation or vitrectomy, but 35.6% were classed as low vision and 8.2% were blind, apparently higher than patients with NPDR (less than 10% and 2%, respectively) and comparable with PDR (34.3% and 15.3%). These results suggested not only a low treatment rate but also a late treatment situation in the population of this study.

Myopic maculopathy was the third leading cause of BCVA-defined visual impairment (18.2%). Our findings were consistent with previous studies on Chinese populations. For example, myopic maculopathy accounted for 7.7% of bilateral blindness in the Beijing Eye Study and 11.0% of the visual impairment in the Handan Eye Study [22, 23]. In this study, only one participant had low vision because of AMD and no one had blindness because of AMD. This finding is different from the consequence found in European diabetic populations. For example, the UK and Denmark, where AMD is the most common cause (42% and 21.9%) of moderate-to-severe visual impairment [20, 24, 27].

Study advantages of this study included a large sample size with a high response rate (91.4%) and standardized protocols. However, some limitations remained in this study. First, we only included subjects with diagnosed DM, hence we could not differentiate causes of visual impairment in the diabetic participants versus those of nondiabetics. Another limitation was that some of the diabetic participants who did not come to the Community Health Centre and did not accept being examined had difficulty moving, partly due to older age coupled with more severe diabetic complications. Most of these participants may have had a high prevalence of sight-threatening diabetic retinopathy and visual impairment. This would lead to a mild underestimation of the prevalence of blindness and low vision in this population.

In summary, this study suggested a high prevalence of low vision and blindness in a group of community people with type 2 diabetes in Northeast China. Our study showed that diabetic retinopathy is the primary leading cause of irreversible low vision and blindness among people suffering with type 2 diabetes. There is an urgent need to improve the screening, prevention, and treatment of diabetic retinopathy in China.

## 5. Conclusions

The prevalence of low vision and blindness of community people with type 2 diabetes in Northeast China is high. Furthermore, the major challenge in decreasing diabetes-related blindness is the early diagnosis and intervention of diabetic retinopathy.

## Figures and Tables

**Table 1:** Prevalence of visual impairment according to visual acuity by gender and age in FS-DIRECT.

Group	*N*	Visual acuity in the better-seeing eye		Visual acuity in the worse-seeing eye	
		Low vision	Blindness	Low vision	Blindness
		<20/60 and ≥20/400	<20/400	<20/60 and ≥20/400	<20/400
		% (95% CI)	% (95% CI)	% (95% CI)	% (95% CI)
*Men*					
30–49	94	8.51 (3.75, 16.08)	3.19 (0.66, 9.04)	11.70 (5.99, 19.97)	6.38 (2.38, 13.38)
50–59	255	5.10 (2.74, 8.56)	1.57 (0.43, 3.09)	11.37 (7.75, 15.92)	4.71 (2.45, 8.08)
60–69	302	7.62 (4.89, 11.21)	0.66 (0.08, 2.37)	22.52 (17.93, 27.65)	6.29 (3.83, 9.65)
70+	167	10.18 (6.04, 15.80)	0 (0.00, 0.00)	22.16 (16.11, 29.22)	11.98 (7.47, 17.89)
Total	818	7.46 (5.75, 9.48)	1.10 (0.50, 2.08)	17.73 (15.17, 20.52)	6.97 (5.32, 8.93)

*Women*					
30–49	72	12.50 (5.88, 22.41)	0 (0.00, 0.00)	33.33 (22.66, 45.43)	2.78 (0.34, 9.68)
50–59	358	12.29 (9.07, 16.15)	0.84 (0.17, 2.43)	16.20 (12.54, 20.43)	6.98 (4.57, 10.14)
60–69	496	11.69 (9.00.14.85)	1.01 (0.33, 2.34)	20.77 (17.28, 24.61)	7.26 (5.13, 9.91)
70+	254	17.32 (12.88, 22.55)	0.39 (0.01, 2.17)	32.28 (26.57, 38.41)	14.96 (10.81, 19.95)
Total	1180	13.14 (11.26, 15.20)	0.76 (0.35, 1.44)	22.63 (20.27, 25.12)	8.56 (7.03, 10.30)

*Total*					
30+	1998	10.81 (9.48, 12.26)	0.90 (0.53, 1.42)	20.62 (18.87, 22.46)	7.91 (6.76, 9.18)
40+	1985	10.68 (9.36, 12.12)	0.91 (0.54, 1.43)	20.55 (18.80, 22.40)	7.96 (6.81, 9.24)
50+	1832	10.86 (9.47, 12.38)	0.82 (0.46, 1.35)	20.58 (18.75, 22.50)	8.19 (6.97, 9.54)
60+	1219	11.65 (9.90, 1 3.58)	0.66 (0.28, 1.29)	23.79 (21.42, 26.28)	9.27 (7.70, 11.04)

FS-DIRECT: Fushun Diabetic Retinopathy Cohort Study.

**Table 2:** Prevalence of visual impairment according to best-corrected visual acuity by gender and age in FS-DIRECT.

Group	*N*	Visual acuity in the better-seeing eye		Visual acuity in the worse-seeing eye	
		Low vision	Blindness	Low vision	Blindness
		<20/60 and ≥20/400	<20/400	<20/60 and ≥20/400	<20/400
		% (95% CI)	% (95% CI)	% (95% CI)	% (95% CI)
*Men*					
30–49	93	3.23 (0.67, 9.14)	1.08 (0.03, 5.85)	3.33 (0.69, 9.43)	2.22 (0.27, 7.80)
50–59	255	1.96 (0.64, 4.52)	1.18 (0.24, 3.40)	4.94 (2.58, 8.47)	2.47 (0.91, 5.30)
60–69	297	0.34 (0.01, 1.86)	0.67 (0.08, 2.41)	8.93 (5.86, 12.90)	2.86 (1.24, 5.55)
70+	162	3.70 (1.37, 7.89)	0 (0.00, 0.00)	10.20 (5.82, 16.27)	5.44 (2.38, 1 0.44)
Total	807	1.86 (1.04, 3.05)	0.74 (0.27, 1.61)	7.24 (5.50, 9.32)	3.16 (2.03, 4.66)

*Women*					
30–49	71	2.82 (0.34, 9.81)	0 (0.00, 0.00)	0 (0.00, 0.00)	2.90 (0.35, 10.08)
50–59	354	1.98 (0.80, 4.03)	0.56 (0.07, 2.03)	6.38 (3.99, 9.59)	2.74 (1.26, 5.13)
60–69	483	1.86 (0.86, 3.51)	0.62 (0.13, 1.80)	11.21 (8.39, 14.59)	3.04 (1.63, 5.14)
70+	245	8.57 (5.38, 12.80)	0 (0.00, 0.00)	14.68 (1.026, 20.09)	8.26 (4.97, 12.74)
Total	1153	3.38 (2.42, 4.60)	0.43 (0.14, 1.01)	9.67 (7.95, 11.63)	4.02 (2.91, 5.40)

*Total*					
30+	1960	2.76 (2.08, 3.58)	0.56 (0.28, 1.00)	8.65 (7.39, 10.04)	3.66 (2.84, 4.63)
40+	1947	2.77 (2.09, 3.60)	0.56 (0.28, 1.01)	8.71 (7.44, 10.11)	3.69 (2.86, 4.66)
50+	1796	2.73 (2.03, 3.59)	0.56 (0.27, 1.02)	9.30 (7.94, 10.81)	3.77 (2.90, 4.81)
60+	1187	3.12 (2.20, 4.27)	0.42 (0.14, 0.98)	11.18 (9.36, 13.22)	4.38 (3.24, 5.78)

FS-DIRECT: Fushun Diabetic Retinopathy Cohort Study.

**Table 3:** Causes of blindness and low vision according to presenting visual acuity.

Causes	Better-seeing eye (WHO standard)		Better-seeing eye (US standard)	
	Blindness^*∗*^	Low vision^＃^	Blindness	Low vision
	No. (%)	No. (%)	No. (%)	No. (%)
Undercorrected refractive error	7 (38.9)	162 (75.0)	74 (77.1)	273 (68.1)
Diabetic retinopathy	5 (27.8)	16 (7.4)	9 (9.4)	31 (7.7)
Proliferative diabetic retinopathy	5 (27.8)	7 (3.2)	8 (8.3)	6 (1.5)
Diabetic maculopathy	0 (0.0)	9 (4.2)	1 (1.0)	25 (6.2)
Cataract	4 (22.2)	24 (11.1)	7 (7.3)	65 (16.2)
Myopic maculopathy	2 (11.1)	10 (4.6)	5 (5.2)	18 (4.5)
Glaucoma	0 (0.0)	1 (0.5)	0 (0.0)	1 (0.2)
Age-related macular degeneration	0 (0.0)	1 (0.5)	0 (0.0)	3 (0.7)
Amblyopia	0 (0.0)	1 (0.5)	1 (1.0)	0 (0.0)
Myopic degeneration	0 (0.0)	0 (0.0)	0 (0.0)	2 (0.5)
Others	0 (0.0)	0 (0.0)	0 (0.0)	2 (0.5)
Uncertain	0 (0.0)	1 (0.5)	0 (0.0)	6 (1.5)
Total	18 (100)	216 (100)	96 (100)	401 (100)

^*∗*^Visual acuity <20/400.^＃^Visual acuity <20/60 and ≥20/400.

**Table 4:** Causes of blindness and low vision according to the best-corrected visual acuity.

Causes	Better-seeing eye (WHO standard)		Better-seeing eye (US standard)	
	Blindness^*∗*^	Low vision＃	Blindness^*∗*^	Low vision＃
	No. (%)	No. (%)	No. (%)	No. (%)
Diabetic retinopathy	5 (45.4)	14 (26.9)	9 (40.9)	31 (24.4)
Proliferative diabetic retinopathy	5 (45.4)	6 (11.5)	8 (36.4)	6 (4.9)
Diabetic maculopathy	0 (0.0)	8 (15.4)	1 (4.5)	25 (19.5)
Cataract	4 (36.4)	24 (46.2)	7 (31.8)	65 (50.8)
Myopic maculopathy	2 (18.2)	10 (19.2)	5 (22.7)	18 (14.1)
Glaucoma	0 (0.0)	1 (1.9)	0 (0.0)	1 (0.8)
Age-related macular degeneration	0 (0.0)	1 (1.9)	0 (0.0)	3 (1.9)
Amblyopia	0 (0.0)	1 (1.9)	1 (4.5)	0 (0.0)
Myopic degeneration	0 (0.0)	0 (0.0)	0 (0.0)	2 (1.6)
Others	0 (0.0)	0 (0.0)	0 (0.0)	2 (1.6)
Uncertain	0 (0.0)	1 (1.9)	0 (0.0)	6 (4.7)
Total	11 (100)	52 (100)	22 (100)	128 (100)

^*∗*^Defined as visual acuity <20/400. ＃Defined as visual acuity <20/60 and ≥20/400.

**Table 5:** Frequency of visual impairment in each individual eye stratified by reference standard diabetic retinopathy (DR) grading according to the best-corrected visual acuity (BCVA).

BCVA	No retinopathy	Mild and moderate NPDR	Severe NPDR	PDR	CSME	Photocoagulation or vitrectomy
	*N* (%)	*N* (%)	*N* (%)	*N* (%)	*N* (%)	*N* (%)
*N*	2272	1117	128	137	127	73
≧20/60	2177 (95.82)	1055 (94.4)	117 (91.4)	69 (050.40)	75 (59.06)	41 (56.2)
<20/60 and ≧20/400	83 (3.65)	60 (5.5)	9 (7.00)	47 (34.3)	46 (36.22)	26 (35.6)
<20/400	12 (0.53)	2 (0.2)	2 (1.60)	21 (15.3)	6 (4.72)	6 (8.2)

NPDR: nonproliferative diabetic retinopathy; PDR: proliferative diabetic retinopathy; CSME: Clinically significant macular edema.

## Data Availability

The dataset used and analysed during the current study is available in the supplementary information file.

## Abbreviations

FS-DIRECT: Fushun Diabetic Retinopathy Cohort Study
DM: Diabetes mellitus
T2DM: Type 2 diabetes mellitus
VA: Visual acuity
BCVA: Best-corrected visual acuity
DR: Diabetic retinopathy
NPDR: Nonproliferative diabetic retinopathy
PDR: Proliferative diabetic retinopathy
DME: Diabetic macular edema.
