# Supplementary material for: The Prevalence and Causes of Visual Impairment in Type 2 Diabetes Mellitus in Northeast China
Source: J Ophthalmol. 2020 Nov 29;2020:5969816. doi: 10.1155/2020/5969816 (PMC7719542; doi:10.1155/2020/5969816)
Supplement: Supplementary Materials — (1) Dataset. [file 5969816.f1.zip › Table 3 Causes of Blindness and Low Vision According to Presenting Visual Acuity.docx]

| **Table 3 Causes of Blindness and Low Vision According to Presenting Visual Acuity** | | | | |
| --- | --- | --- | --- | --- |
|  | Better-Seeing Eye (WHO standard) | | Better-Seeing Eye (US standard) | |
|  | Blindness* | Low Vision^＃^ | Blindness | Low Vision |
| Causes | No. (%) | No. (%) | No. (%) | No. (%) |
| Undercorrected refractive error | 7（38.9） | 162（75.0） | 74（77.1） | 273（68.1） |
| Diabetic retinopathy | 5(27.8) | 16(7.4) | 9(9.4) | 31(7.7) |
| Proliferative diabetic retinopathy | 5(27.8) | 7(3.2) | 8(8.3) | 6(1.5) |
| Diabetic maculopathy | 0(0.0) | 9(4.2) | 1(1.0) | 25(6.2) |
| Cataract | 4(22.2) | 24(11.1) | 7(7.3) | 65(16.2) |
| Myopic maculopathy | 2(11.1) | 10(4.6) | 5(5.2) | 18(4.5) |
| Glaucoma | 0(0.0) | 1(0.5) | 0(0.0) | 1(0.2) |
| Age-related macular degeneration | 0(0.0) | 1(0.5) | 0(0.0) | 3(0.7) |
| Amblyopia | 0(0.0) | 1(0.5) | 1(1.0) | 0(0.0) |
| Myopic degeneration | 0(0.0) | 0(0.0) | 0(0.0) | 2(0.5) |
| Others | 0(0.0) | 0(0.0) | 0(0.0) | 2(0.5) |
| Uncertain | 0(0.0) | 1(0.5) | 0(0.0) | 6(1.5) |
| total | 18(100) | 216 (100) | 96(100) | 401（100） |
| *Defined as visual acuity <20/400. | | | | |
| ＃Defined as visual acuity <20/60 and ≥20/400. | | | | |
